# Preparation of Molecularly Imprinted Mesoporous Materials for Highly Enhancing Adsorption Performance of Cytochrome C

**DOI:** 10.3390/polym10030298

**Published:** 2018-03-10

**Authors:** Zhiling Li, Ping Guan, Xiaoling Hu, Shichao Ding, Yuan Tian, Yarong Xu, Liwei Qian

**Affiliations:** 1 Key Laboratory of Space Applied Physics and Chemistry of Ministry of Education, Department of Applied Chemistry, School of Nature and Applied Science, Northwestern Polytechnical University, Xi’an 710072, China; lizhiling1993@mail.nwpu.edu.cn (Z.L.); huxl@nwpu.edu.cn (X.H.); dingshichao1992@163.com (S.D.); summer940324@163.com (Y.T.); xuyarong@mail.nwpu.edu.cn (Y.X.); 2 College of Bioresources Chemical and Materials Engineering, Shaanxi University of Science and Technology, Xi’an 710021, China

**Keywords:** amphiphilic ionic liquids, molecularly imprinted mesoporous silica, epitope imprinting, Cytochrome c

## Abstract

Molecularly imprinted mesoporous materials (MIMs) were synthesized to improve the adsorption performance of Cytochrome c (Cyt c) by using an imidazolium-based amphiphilic ionic liquid 1-octadecyl-3-methylimidazolium chloride (C_18_MIMCl) as surfactant in aqueous solution via the epitope imprinting approach. The surface-exposed C-terminus nonapeptide of Cyt c (residues 96–104, AYLKKATNE) was utilized as the imprinted template. The nitrogen adsorption-desorption, thermo-gravimetric analysis, and transmission electron microscopy verified the successful preparation of MIMs with ordered mesoporous structure. The adsorption isotherm studies showed that the obtained MIMs exhibited superior adsorption capacity toward Cyt c of 86.47 mg·g^−1^ because of the high specific surface areas of 824 m^2^·g^−1^, and the appropriate pore size promoted the mass transfer of Cyt c, causing a rapid adsorption equilibrium within 20 min. Furthermore, these MIMs still remained excellent selectivity and recognition ability according to the selective as well as the competitive adsorption studies, suggesting that the molecularly imprinted mesoporous materials is expected to be used in the field of highly efficient separation and enrichment of proteins.

## 1. Introduction

Along with the wide development of bioseparetion, biosensing or biomedical materials, the separation and identification of proteins have drawn great attention recently [1]. In the past, the detection of proteins was almost always based on the antigen-antibody reactions, however, the high cost and structural sensitivity somewhat hindered the recycling of antibodies [2]. Molecular imprinting technology (MIT) is an emerging separation method that could achieve the fast and high selective separation of the target molecule with an inexpensive and stabilized biomaterial [3,4]. Nowadays, numerous molecularly imprinted polymers (MIPs) were developed and applied in small template molecules, while the imprinting of proteins still has limitations because of the huge molecular size, flexible conformation, and complex structures [5,6]. In order to overcome these challenges, researchers have proposed epitope imprinting [7,8], which is based on using a specific fragment of protein as template. Those obtained MIPs by epitope imprinting had the ability to recognize the whole protein [9,10]. However, along with the low conformation destructiveness of the epitope imprinting system, some new problems emerges, i.e., how to get enough surface area to reach a high total amount of the recognized target proteins.

Ordered mesoporous silica materials [11,12,13] with a large surface area was a desired substrate to increase the imprinted sites amounts and enhance the adsorption capacity of target molecule. By making use of the interactions between the surfactant and template, it could be ensured that a portion of the template remained at the surface of micelles [14]. Then, functional monomers and a cross-linker could be copolymerized in the presence of the template protein. After the elution of template, one-step interfacial molecularly imprinted mesoporous silica was obtained. Chen et al. prepared an adenosine monophosphate (AMP)-imprinted mesoporous silica nanoparticle materials (MSNs) with the pore diameter of 2.3 nm and the BET surface area of 833 m^2^·g^−1^ [15]. The obtained MIPs significantly improved the imprinting efficiency and binding properties toward AMP. Moreover, the tunable pore sizes of the mesoporous materials provided an enough space for the mass transfer of biomacromoleucles, such as proteins [16]. The critical factor is developing a surfactant to controllably grow a mesoporous with easily accessible mesopore, so as to meet the requirements in immobilization of template. Unfortunately, the pore size of some mesoporous support materials by using trimethylammonium bromide (CTAB), as the surfactant is less than the mean size of proteins [17,18], which would reduce the adsorption performance of imprinted polymers for proteins. Therefore, suitable alternatives to CTAB, which not only act as a surfactant, but also generate suitable sizes of mesopores and channels for the diffusion of proteins, are of great importance. 

Amphiphilic ionic liquids (ILs) consisting of an imidazolium cation and a hydrophobic long alkyl chain along with a kosmotropic anion could direct the formation of mesophase in the synthesis system and produce well-ordered mesoporous materials [19,20,21]. The mesopore size can be tuned by varying the length of the alkyl chain and the anion [22]. In addition, the researchers have shown that ILs with an imidazolium cation as the polar group could provide multiple interactions in immobilizing template molecules [23,24,25], which ensured the adsorption capacity of MIPs. Recently, our group reported an interfacial molecularly imprinted microspheres by using imidazolium-based amphiphilic ionic liquid (IL) as emulsifier and suggested that the amphiphilic IL could immobilize protein at the liquid-water interface by intermolecular forces [26]. Therefore, taking into consideration the potential of ILs to adjust mesopore diameter and provide multiple interactions, designing an imidazolium-based amphiphilic IL as surfactant would improve the adsorption performance of MIPs.

Based on the above reasons, in order to improve the adsorption performance of MIPs, molecularly imprinted mesoporous materials (MIMs) were synthesized to enrich and detect Cytochrome c (Cyt c) by using the surface-exposed C-terminal fragment of Cyt c (AYLKKATNE) as template. Amphiphilic IL 1-octadecyl-3-methylimidazolium chloride (C_18_MIMCl) was utilized as surfactant to control the mesopore size and immobilize template. The morphologies and structures of MIMs were characterized by transmission electron microscope (TEM), Brunauer–Emmett–Teller (BET) nitrogen adsorption and thermo-gravimetric analysis (TGA). Furthermore, the adsorption properties, specific recognition ability, and reusability of MIMs for Cyt c were systematically investigated.

## 2. Experimental

### 2.1. Materials

1-Chlorooctadecane were obtained from Macklin (Shanghai, China). NaOH, HCl, methanol, ethanol and tetrahydrofuran (THF) were provided by Kermel (Tianjin, China). The monomers 3-Aminopropyltriethoxysilane (APTES, Aldrich, Shanghai, China) and 1-Propyltrimethoxysilane-3-methylimidazolium chloride (PTESMIC, Cheng Jie, Shanghai, China) were used without further purification. Tetraethyl orthosilicate (TEOS, 99%) and 1-Methylimidazole were obtained from Aladdin (Shanghai, China). Cytochrome c (Cyt c, *M*_w_ = 12.4 kDa, p*I* = 11.2) was purchased from Macklin (Shanghai, China). Lysozyme (Lyz, *M*_w_ = 13.4 kDa, p*I* = 9.6) and Ovalbumin (OVA, *M*_w_ = 45.0 kDa, p*I* = 4.7) were provided by MP Biomedicals. Bovine hemoglobin (BHb, *M*_w_ = 64 kDa, p*I* = 6.9) and Bovine serum album (BSA, *M*_w_ = 67 kDa, p*I* = 4.9) were purchased from Sigma-Aldrich. The peptide chains, including C-terminal fragment of Cyt c (AYLKKATNE), were synthesized by KareBay (Ningbo, China).

### 2.2. Synthesis of Amphiphilic Ionic Liquid C_18_MIMCl

**Figure 1:**
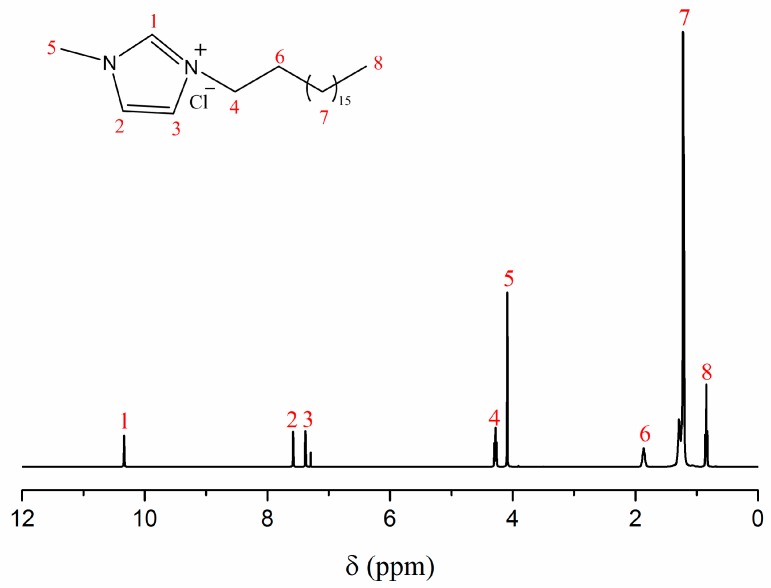
^1^H NMR spectrum of C_18_MIMCl in CDCl_3_.

The synthesis of C_18_MIMCl was performed according to a reported route [27]. As a typical synthesis process, 1-Methylimidazole (0.1 mol) was mixed with 1-Chlorooctadecane (0.1 mol). The above-mixture was put into a 250 mL flask with refluxing at 90 °C for 24 h. When cooling to room temperature, a kind of white solid was obtained. Then, the product was further purified by recrystallization in THF. After washing several times with THF, the resulting white powder was collected by filtration and dried under vacuum at room temperature. The structure of C_18_MIMCl is shown in Figure 1.

^1^H NMR (400 MHz, CDCl_3_): δ 10.34 (s, 1H), 7.58 (s, 1H), 7.38 (s, 1H), 4.28 (t, *J* = 7.4 Hz, 2H), 4.09 (s, 3H), 1.92–1.80 (m, 2H), 1.35–1.10 (m, 22H), 0.84 (t, *J* = 6.8 Hz, 3H).

### 2.3. Preparation of Molecularly Imprinted Mesoporous Materials (MIMs)

Firstly, the C_18_MIMCl and NaOH (0.17 g) were dissolved in distilled water (288 mL) simultaneously. Then, the solution was transferred into a 500 mL three-necked round-bottomed flask and was stirred at 80 °C for 15 min at a speed of 700 rpm. Subsequently, 15 mg of template (C-terminal peptides of Cyt c, as AYLKKATNE), TEOS (2.5 mL), APTES (0.1 mL) and PTESMIC (0.38 g) were added dropwise to the mixture and then reacted at 400 rpm for 2 h. Finally, the obtained white powder was washed with ethanol for several times and evaporated under vacuum at 40 °C for further use. Additionally, the complete removal of C_18_MIMCl and nonapeptide from MIMs was implemented with Soxhlet extraction method by mixing HCl (36%, 2.5 mL) and methanol (150 mL). After that, these imprinted materials (MIMs) were dried under vacuum at 40 °C for 72 h to remove residual solvent within the mesoporous materials.

In a parallel, non-imprinted mesoporous materials (NIMs) was prepared by the same procedure, but in the absence of template nonapeptide.

### 2.4. Characterization

^1^H NMR spectra of ILs was obtained from a Varian spectrometer at 400 MHz using CDCl_3_ as the solvent. The morphologies and structures of MIMs and NIMs were determined by transmission electron microscopy (TEM, Talos F200X G2). The surface areas and the pore size distributions were calculated by the Brunauer-Emmett-Teller (BET) and Barrett-Joyner-Halenda (BJH) methods. The content of functional monomers was measured by thermo-gravimetric analysis (TGA, Q50, TA Instruments, Shanghai, China). For the detection of template peptides (AYLKKATNE), Cyt c, and comparative protein, UV-vis spectra were obtained using a UV-2550 spectrophotometer (Shimadzu). Sodium dodecyl sulfate polyacrylamide gel electrophoresis (SDS-PAGE) was performed using a DYY-6C electrophoresis system (Beijing Liuyi Instrument Plant, Beijing, China).

### 2.5. Isothermal Rebinding and Dynamic Adsorption

The dried MIMs/NIMs (50 mg) were dispersed in 5 mL buffer solution (pH = 7.4) with different concentrations of Cyt c (from 0.2 to 1.4 mg·mL^−1^). After incubation with shaking at room temperature for 24 h, the supernatant was further filtrated through a 0.1 μm polyethersulfone syringe filter. Then, the concentration of Cyt c in the supernatant was determined using a UV-vis spectrophotometer.

The adsorption dynamic studies were performed by detecting the adsorption amount of MIMs/NIMs for Cyt c at regular incubation time. First, 50 mg dried MIMs/NIMs were incubated in 5mL phosphate buffer (pH = 7.4) containing 1.0 mg·mL^−1^ of Cyt c at 25 °C for 24 h. Then, the concentration of residual Cyt c solution was measured by UV at different incubation time.

The adsorption amount *Q*_e_ (mg·g^−1^) of MIMs/NIMs for protein was evaluated according to Equation (S1), and the imprinting factor (*IF*) was calculated by Equation (S2).

### 2.6. Selectivity and Competitive Adsorption Experiments

In order to estimate the selectivity recognition of MIMs for Cyt c, 50 mg dried MIMs/NIMs were incubated in 5 mL phosphate buffer solution (pH = 7.4) containing target protein Cyt c and protein analogue (Lyz, BHb and BSA) separately with a concentration of 1.0 mg·mL^−1^, and the mixture was shaken at 25 °C for 24 h. The amount of each protein that was adsorbed by MIMs/NIMs was determined by measuring the UV absorbance.

The competitive adsorption experiment was conducted with adding 50 mg dried MIMs/NIMs into 5 mL phosphate buffer (pH = 7.4) containing both Cyt c and its competitor at 25 °C with each protein concentration of 1.0 mg·mL^−1^. After incubation for 24 h under gentle shaking, MIMs/NIMs were removed from the solution by centrifugation and treated first with 5 mM NaCl to rinse the weakly adsorbed proteins on the mesoporous surface of MIMs/NIMs and then with 0.5 M NaCl to elute the specifically adsorbed proteins. The specifically adsorbed proteins were desalted by using dialysis tubing with a molecular cutoff of 500 Da, and were then freeze-dried for 24 h. Thereafter, the obtained protein was dissolved in 5 mL phosphate buffer (pH = 7.4) and was analyzed by SDS-PAGE using 12.5% polyacrylamide separating gel and 5% polyacrylamide stacking gel.

### 2.7. Reusability Experiments

Reusability is one of an important property of MIPs in practical applications. To evaluate the reusability of the MIMs and NIMs, 50 mg dried MIMs/NIMs were suspended in 5 mL protein solution (pH = 7.4) with a concentration of 1.0 mg·mL^−1^. After adsorption and centrifugation, the concentration of Cyt c residue was determined by UV-vis spectrophotometer. Thereafter, the recovered MIMs were eluted for the removal of the template and were then further reused for subsequent adsorption-desorption cycles. The reusability adsorption experiments were performed six times at ambient temperature by the same batches of MIMs.

## 3. Results and Discussion

### 3.1. The Effect of Alkyl Chain Length of ILs on Mesoporous Diameter

The appropriate mesopore size could promote the mass transfer and recognition of protein [28]. Cytochrome c (Cyt c), a structurally robust heme protein with dimensions of ~2.6 nm × 3.2 nm × 3.3 nm, was used as the target protein to explore the adsorption and recognition process of proteins that were entering the imprinted sites [29]. In order to explore the suitable pore size for recognition Cyt c, three kinds of ILs were synthesized as surfactants to prepare three mesoporous silica nanoparticle materials (MSNs), which were 1-octyl-3-methylimidazolium chloride (C_8_MIMCl)-MSNs, 1-tetradecyl-3-methylimidazolium chloride (C_14_MIMCl)-MSNs, and 1-octadecyl-3-methylimidazolium chloride (C_18_MIMCl)-MSNs, respectively. The detailed synthesis process of C_8_MIMCl, C_14_MIMCl, and C_n_MIMCl-MSNs were explained in supplementary materials. The structure of C_8_MIMCl and C_14_MIMCl are shown in Figures S1 and S2. The synthetic schematic of C_n_MIMCl-MSNs with different pore size was displayed in Scheme S1. Firstly, the ionic liquid was dissolved in an aqueous solution to form micelles, and then the mesoporous material was synthesized by a one-step sol-gel method.

The TEM images of C_n_MIMCl-MSNs were showed in Figure S3. It was employed to investigate the morphologies of C_n_MIMCl-MSNs. The obtained C_n_MIMCl-MSNs exhibited a spherical shape with ordered mesopores, which confirmed that the C_n_MIMCl-MSNs were prepared successfully. The average pore diameter and specific surface areas of MSNs were conducted by nitrogen adsorption-desorption. As shown in Table 1, the BJH average pore diameters of these materials increased as the alkyl chain of the ILs lengthened. The pore size varied approximately in the range of 1.80–3.60 nm with the varied carbon chain lengths of the used ILs. It was because that the concentrations of ILs were higher than there critical micelle concentration (CMC) in water system, hydrophobic alkyl chain will gather to form the micelles with different diameters. When the micelle rods were removed, mesoporous channels with different diameters were obtained. When compared with C_8_MIM-MSNs and C_14_MIM-MSNs, C_18_MIM-MSNs possessed the most optimal and suitable pore diameter for the mass transfer of Cyt c in the pore. Therefore, using the amphiphilic IL C_18_MIMCl as surfactant could provide satisfied pore diameter for adsorption of Cyt c by MIMs in rebinding tests.

### 3.2. Preparation and Characterization of MIMs and NIMs

**Scheme 1:**
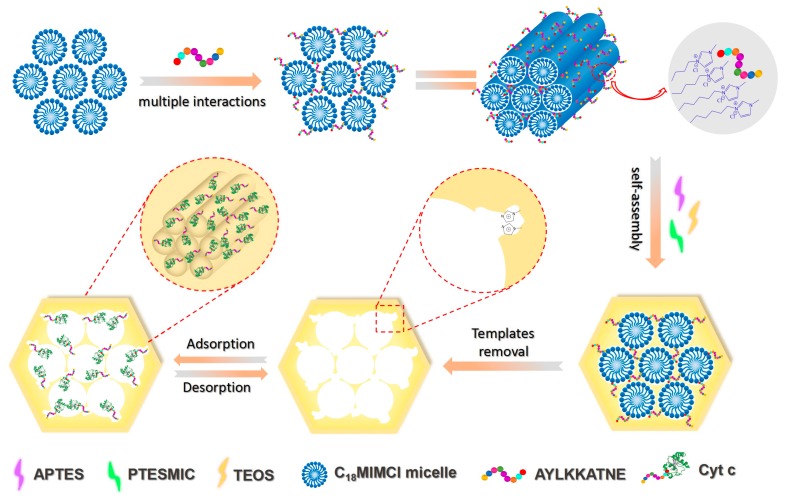
Schematic illustration of the synthesis procedure of MIMs and the selective process for the enrichment of Cytochrome c (Cyt c) using MIMs.

The MIMs was prepared by combining the epitope imprinting technique and sol-gel method (Scheme 1). Based on above pore size experiment results, the amphiphilic IL with an imidazole ring (C_18_MIMCl) was utilized as surfactant. The nonapeptide template was anchored on the surface of C_18_MIMCl micelle rods through multiple interactions [30] generating surfactant-template complexes. After removal of the surfactant-template complexes, all of the imprinted sites were formed on the surface of mesoporous channels. The obtained MIMs could be an ideal sorbent for the selective enrichment of Cyt c.

**Figure 2:**
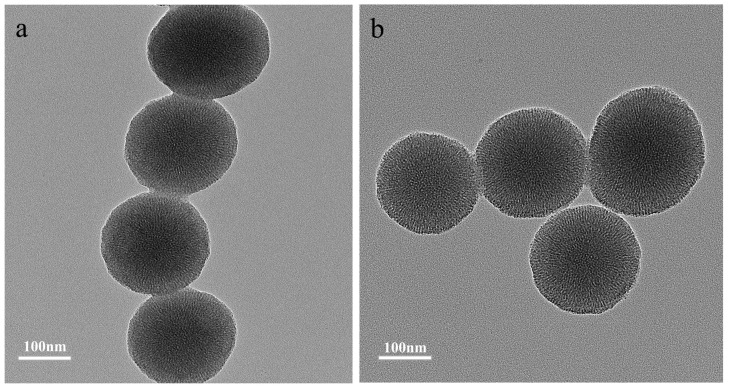
Transmission electron microscope (TEM) images of MIMs (**a**) and NIMs (**b**).

The TEM images of MIMs and NIMs were showed in Figure 2a,b. It is noted that the nonapeptide-imprinted and non-imprinted mesoporous materials displayed a fibrous porous morphology that is directed by C_18_MIMCl. Both the MIMs and NIMs were nearly spherical in shape with an average diameter about 200 nm. The smaller spherical size and regularly ordered mesopores produced a large specific surface area of imprinted mesoporous materials, which will be beneficial to the adsorption performance of MIMs. The average size of MIMs was investigated by dynamic light scattering (DLS). As shown in Figure S4, the size distribution of MIMs was about 200 nm, which was consistent with TEM.

**Figure 3:**
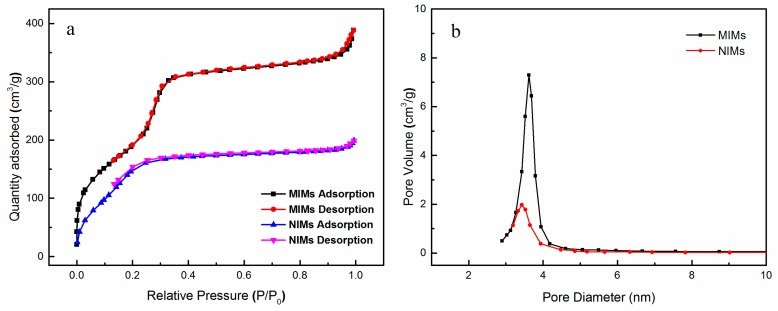
N_2_ adsorption–desorption isotherms (**a**) and pore size distribution (**b**) for molecularly imprinted mesoporous materials (MIMs) and non-imprinted mesoporous materials (NIMs).

The N_2_ adsorption-desorption isotherms and pore size distribution of MIMs and NIMs were also performed. As shown in Figure 3a,b, the pore diameter of MIMs and NIMs were measured to be 3.62 and 3.43 nm, and the specific surface areas were calculated to be 824 and 665 m^2^·g^−1^, respectively. According to the mechanism of mesoporous formation, these differences may be attributed to the imprinted cavities mostly embedding in the surface of mesoporous channel of MIMs, which made a local expansion of the regular mesopores. Therefore, the average pore size and specific surface areas were increased. Additionally, the appropriate pore diameter and the larger surface areas of MIMs will contribute to the mass transfer of Cyt c in the mesopores.

**Figure 4:**
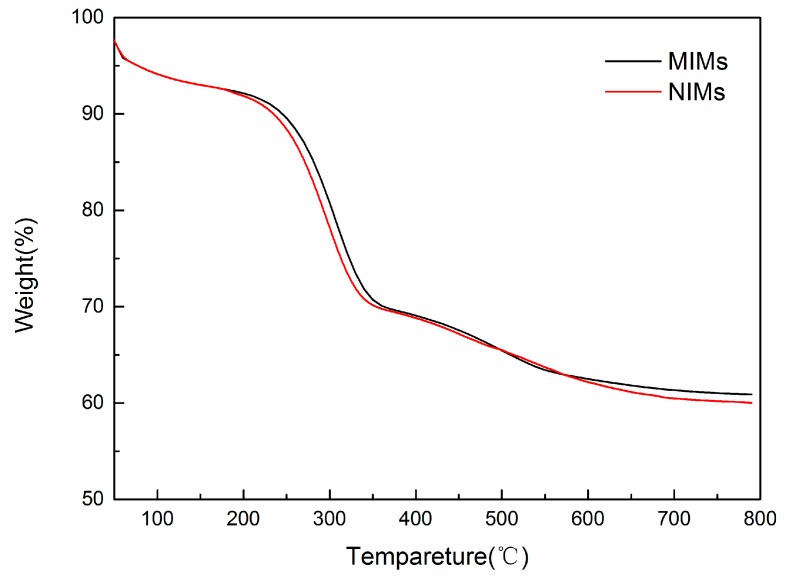
Thermo-gravimetric analysis (TGA) curves of MIMs and NIMs.

The solid content of MIMs and NIMs were studied by TGA, and the results were showed in Figure 4. All materials had slightly decline before temperature rising to 200 °C, which were the weight loss of adsorbed moisture and surface organogels or inorganic gels. When the temperature was increased above 200 °C, the loss of MIMs and NIMs were due to the decomposition of APTES and PTESMIC. This phenomenon demonstrated that the heat losses of MIMs and NIMs were almost identical, indicating that the degree of polymerization of both was consistent. Therefore, the measured value of adsorption property for Cyt c would not be affected by differences in polymerization.

### 3.3. Adsorption Properties of MIMs and NIMs

**Figure 5:**
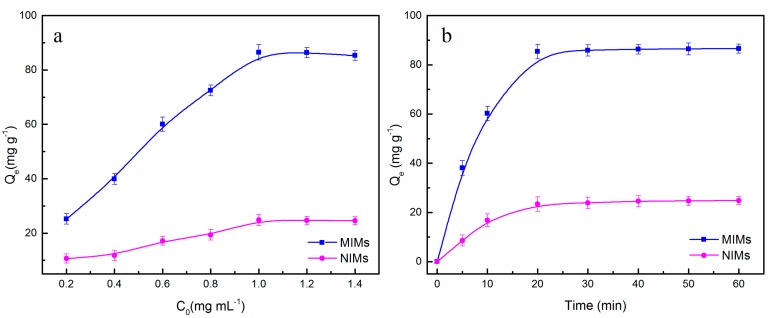
Adsorption isotherm of MIMs and NIMs with Cyt c (**a**); Adsorption dynamic curves of MIMs and NIMs with Cyt c (**b**).

For investigating the imprinting efficiency and binding properties of MIMs for target protein Cyt c, adsorption isotherm experiments were conducted in Cyt c solutions with a certain concentration. From the Figure 5a, it is easy to observe that the adsorption capacity enhanced with an increasing Cyt c concentration. The adsorption equilibrium capacity *Q*_e_ of MIMs increased sharply with the initial concentration of Cyt c, and then saturated over 1.0 mg·mL^−1^, while the maximum adsorption capacity of Cyt c reached 86.47 mg·g^−1^. However, the *Q*_e_ of NIMs for Cyt c was much lower than that of MIMs and the maximum adsorption amount was only 24.85 mg·g^−1^, which showed that MIMs had a higher binding ability than that of NIMs. The relative high adsorption capacity might be attributed to the multiple interactions (such as hydrogen bonding, π–π stacking, ion-ion electrostatic, and van der Waals interactions, etc.) afforded by C_18_MIMCl [31]. Particularly, the ion-ion electrostatic interaction provided by imidazolium group of C_18_MIMCl and PTESMIC is much stronger than the other interactions [32], which could induce the aggregation of oppositely charged nonapeptide around the micelles and facilitated the formation of more imprinting sites on the mesoporous surface. The maximum *IF* was calculated to be 3.48, indicating that the MIMs had better specific recognition when compared with NIMs, because the specific recognition cavities were generated on the surface of mesoporous channels of MIMs in the imprinting process.

The experimental data was fitted by different isotherm models, including Langmuir model (Equation (S3)) and Freundlich model (Equation (S4)). The Langmuir model assumes that adsorption took place on a homogeneous surface with identical active sites and uniform energies, while the Freundlich model assumes that adsorption occurred on a heterogeneous surface with the exponential distribution of active sites and energies [33]. The calculated parameters were listed in Table 2. As shown in Table 2, the Langmuir model gave a better fitting in the range of concentrations than that of Freundlich model via the correlation coefficient (*R*^2^) value, which showed that the adsorption of MIMs to the target protein Cyt c is monolayer adsorption. Because the MIMs was prepared by using nonapeptide as template via epitope imprinting approach to separate and recognize Cyt c. The pore size of the imprinted sites is slightly larger than the average mesopore size around 3.62 nm. Therefore, the MIMs was more likely to form a single specific adsorption for Cyt c at the imprinted sites in a limited space.

For NIMs, the Freundlich model and the Langmuir model yielded a similar fit to the equilibrium data in terms of the correlation coefficients (*R*^2^) values, which was related to the random arrangements of functional groups and the absence of specific recognition sites in NIMs.

To determine the rate of adsorption and the binding capacities as a function of time, the adsorption dynamics of MIMs and NIMs were carried out by using 1.0 mg·mL^−1^ Cyt c buffer solution at 25 °C. As shown in Figure 5b, both MIMs and NIMs showed a rapid increase during the first 20 min, and then the adsorption process reached equilibrium after 20 min. Obviously, the adsorption rate and adsorption capacities of MIMs for Cyt c in the initial phase were higher than that of NIMs. When compared with the previous works of our group [26,34], the imprinted mesoporous materials showed a faster adsorption rate for target protein. It is attributed to the specific recognition sites increased by the large internal surface areas of mesoporous silica, and highly accessible sites locating on the wall of the mesoporous, which also be responsible for the excellent adsorption performance of MIMs. Moreover, it also implied that the imprinted mesoporous materials prepared by epitope imprinting method can achieve rapid and specific adsorption of Cyt c.

The controlling mechanism of dynamic binding process was further investigated by the pseudo-first-order and pseudo-second-order kinetic models via Equations (S5) and (S6). In general, the pseudo-first-order model indicates that the occupation rate of adsorption sites is proportional to the number of unoccupied sites, whereas the pseudo-second-order model assumes that the adsorption rate is controlled by chemical adsorption [35]. According to the correlation coefficient (*R*^2^) value in Table 3, the pseudo-second-order kinetic model showed a better correlation with the MIMs, which demonstrated that chemical adsorption might be the rate-limiting step in the process of recognition, indicating that imprinted sites were actually formed and that they are functionally and sterically complementary affinity to nonapeptide fragment on Cyt c.

### 3.4. Selectivity Study

**Figure 6:**
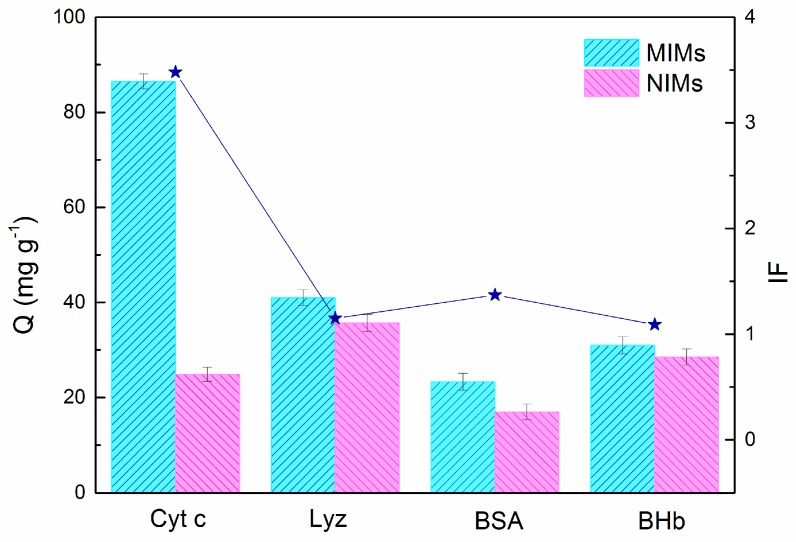
Selectivity of MIMs and NIMs in the adsorption of proteins.

The selective adsorption experiment of MIMs and NIMs for different proteins was performed in buffer solution (pH = 7.4) with a feed concentration of 1.0 mg·mL^−1^. Three proteins of BSA, BHb, and Lyz were chosen as reference proteins for Cyt c. As presented in Figure 6, the adsorption capacities of MIMs for BSA and BHb were much lower than that for Cyt c. Owing to the conformational differences among Cyt c, BSA, and BHb, the imprinted cavities of MIMs more matched with the C-terminal nonapeptide of Cyt c. However, the adsorption capacities of MIMs for another reference protein, Lyz, was slightly higher than that for BSA and BHb because Lyz has the close isoelectric point (p*I*) and molecular wight (*M*_w_) with Cyt c and can enter the mesopores causing high non-specific adsorption capacity. The corresponding imprinting factor *IF* of MIMs for BSA, BHb and Lyz were also reported in Figure 6. *IF*s of these proteins decreased in the order of Cyt c > BSA > BHb > Lyz, which indicated that the imprinted sites were closely match with Cyt c in the interaction sites and the three-dimensional space.

**Figure 7:**
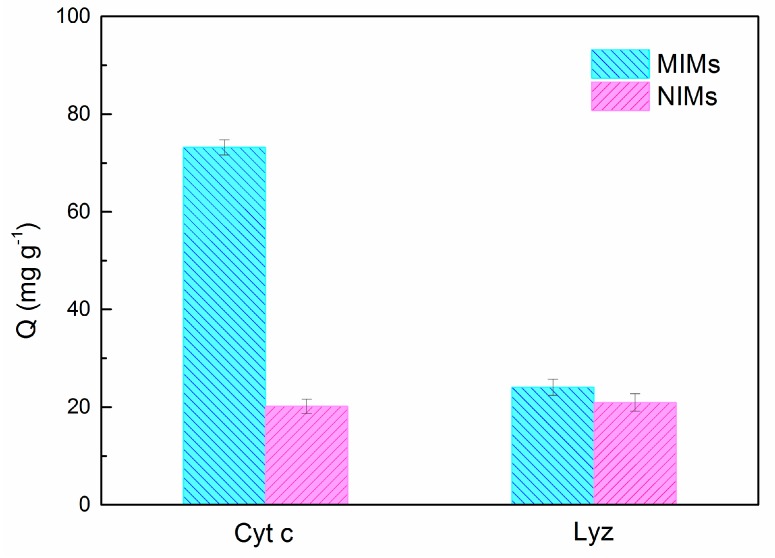
Selective binding of MIMs and NIMs for Cyt c and Lyz, respectively.

The above selective adsorption experiment showed that Lyz is a potential competitor to Cyt c. In order to further investigate the selective recognition of MIMs for Cyt c, the binary competitive adsorption experiments were performed in complex samples containing Cyt c and Lyz with concentration of 1.0 mg·mL^−1^. As presented in Figure 7, the adsorption capacity of MIMs for Cyt c was higher than that of Lyz, suggesting that the functional groups that were located at imprinted cavities could offer functionally complementary affinity toward Cyt c in above complex environment, as compared with reference protein Lyz, and thus imprinted cavities had excellent specificity for Cyt c.

### 3.5. Competitive Batch Rebinding Tests

**Figure 8:**
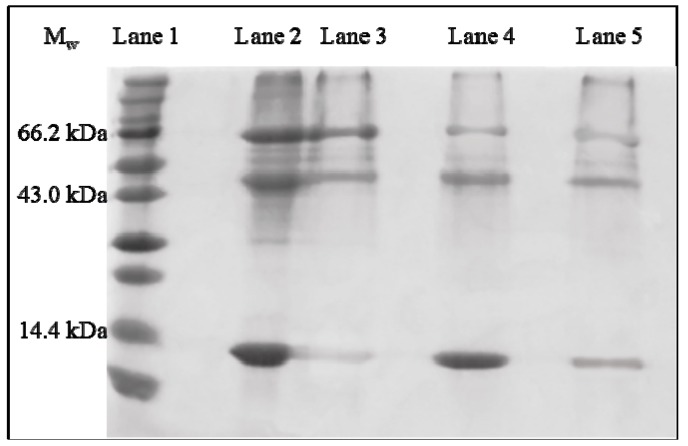
Sodium dodecyl sulfate polyacrylamide gel electrophoresis (SDS-PAGE) results of competitive adsorption experiment. Lane 1, protein molecular weight marker; Lane 2, solution of mixture of Bovine serum album (BSA), Ovalbumin (OVA), and Cyt c; Lane 3, the protein in the mixed solution after adsorption by MIMs; Lane 4, the protein eluted from MIMs; Lane 5, the protein eluted from NIMs.

To further illustrate the specific recognition property of the MIMs for the Cyt c, BSA, and OVA were used as the competitive proteins in the buffer solution. As shown in Figure 8, lane 2 presented BSA, OVA and Cyt c sequentially with each concentration of 1.0 mg·mL^−1^. After the adsorption by MIMs, the content of Cyt c in the mixed solution decreased significantly (lane 2 to lane 3). The intensity of the template Cyt c band eluted from MIMs (lane 4) was higher than that of corresponding NIMs (lane 5), revealing that the MIMs had a better ability to specifically recognize Cyt c. Moreover, the bands of BSA and OVA in lane 4 were shallower than the lane 2, suggesting that a little amount of BSA and OVA was captured by MIMs. In general, MIMs could selectively capture and effectively enrich Cyt c from the complex protein mixture.

### 3.6. Comparison of Imprinting Methods for Cyt C

To check the superior adsorption performance of MIMs with amphiphilic ILs as the surfactant to control pore size and immobilize template, we investigated the adsorption capacity and the equilibrium rate of MIMs for Cyt c in other related researches. As shown in Table 4, owing to advantages of the high specific surface areas of mesoporous silica and the epitope imprinting method, this new imprinting strategy could obtain much higher binding capacity toward Cyt c than that of others imprinted polymers [36,37,38]. From the comparison of the adsorption rate, we could conclude that this approach still displayed the rapid adsorption equilibrium on the basis of a superior adsorption capacity. That further demonstrated the MIMs have the potential to be applied in the field of molecular imprinting.

### 3.7. Reusability

**Figure 9:**
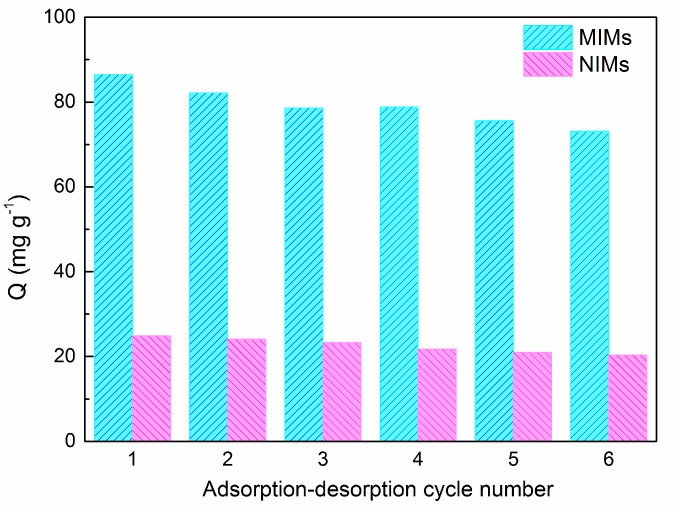
Reusabilities of MIMs and NIMs.

Reproducibility is one of an important criterion to evaluate the property of imprinted materials in the practical purposes. Therefore, the reusability of MIMs for six adsorption-desorption cycles was investigated in Cyt c solution (pH = 7.4) with a feed concentration of 1.0 mg**·**mL^−1^. As shown in Figure 9, the adsorption capacity of MIMs decreased slightly after each elution step, and 13.43 mg**·**g^−1^ adsorption capacity was lost after six cycles. In order to observe the decreased adsorption amount of each elution step more directly, the statistical analysis of the reusability of MIMs was showed in Table S1. Because some recognition sites in the mesopore might be destroyed or blocked during the washing procedure, which were not helpful to recognize the Cyt c anymore. When compared to MIMs, there was little decrease in case of adsorption capacity of NIMs because no specific adsorption sites were located in them.

## 4. Conclusions

In this work, we have synthesized molecularly imprinted mesoporous materials (MIMs) via epitope imprinting and the sol-gel method. Amphiphilic IL C_18_MIMCl was used as the surfactant to direct the formation of mesopores and immobilize template. The multiple interactions between C_18_MIMCl and nonapeptide could ensure the positioning of nonapeptide at the micelle interface, which was primary for the generation of imprinted cavities at the surface of mosoporous channels. The prepared imprinted mesoporous materials provided a suitable pore diameter to promote the mass transfer of Cyt c spaciously. The large specific surface areas of MIMs guaranteed enough recognition sites. Due to these advantages, the MIMs not only exhibited high adsorption capacity (86.47 mg**·**g^−1^) and rapid adsorption equilibrium (20 min), but also displayed superior specificity and selectivity toward Cyt c by analyzing the selectivity and competitive adsorption experiments. Furthermore, the reusability makes it an ideal candidate to be applied in protein purification and separation science. We concluded that the strategy of combining molecular imprinting mesoporous materials and amphiphilic ILs can yield smart imprinted materials with rapid adsorption rate, excellent adsorption performance, and high specificity and selectivity, thus advancing the field of protein imprinting one step further.

## Figures and Tables

**Table 1:** Nitrogen Sorption Data of C_n_MIM-mesoporous silica nanoparticle materials (MSN) Materials.

Ionic Liquids	BET Surface Areas	Pore Volume	BJH Average Pore
(ILs)	(m^2^/g)	(cm^3^/g)	Diameter (nm)
C_8_MIMCl	1031	0.673	1.85
C_14_MIMCl	692	0.662	2.23
C_18_MIMCl	795	0.810	3.58

**Table 2:** Isotherm model and constants of MIMs, NIMs for Cyt c.

Isotherm Model	Langmuir			Freundlich		
Materials	*Q*_max_ (mg·g^−1^)	*K*_L_ (mg·mL^−1^)	*R*^2^	*K*_F_ (mg·g^−1^)	*n*	*R*^2^
MIMs	156.05	1.0192	0.9632	77.29	1.6931	0.9340
NIMs	38.61	1.3998	0.9256	22.12	1.9729	0.9291

**Table 3:** Kinetic constants of the pseudo-first-order and pseudo-second-order.

		Pseudo-First-Order			Pseudo-Second-Order		
Materials	*Q*_e,e_^a^ (mg·g^−1^)	*Q*_e,c_^b^ (mg·g^−1^)	*K*_1_ (min^−1^)	*R*^2^	*Q*_e,c_^b^ (mg·g^−1^)	*K*_1_ (g mg^−1^·min^−1^)	*R*^2^
MIMs	86.47	87.86	0.1209	0.9784	101.10	0.0015	0.9948
NIMs	24.85	25.11	0.1048	0.9753	29.71	0.0040	0.9919

^a^ *Q*_e,e_ is the experimental value of *Q*_e_; ^b^ *Q*_e,c_ is the calculated value of *Q*_e_.

**Table 4:** Comparison of different methods of imprinting Cyt c.

Carrier	Imprinting Strategies	Recongation Interactions	Adsorption Equilibrium	*Q* (mg·g^−1^)	Ref.
BC nanofibers	Protein imprinting	metal ion coordination	10 min	36.4	[36]
Fe_3_O_4_@SiO_2_	Epitope imprinting	Multiple	2 h	67.6	[37]
Organic polymers	Protein imprinting	Hydrogen bond	4 h	67.4	[38]
Mesoporous silica	Epitope imprinting	Multiple	20 min	86.47	This method

## Supplementary Materials

The following are available online at http://www.mdpi.com/2073-4360/10/3/298/s1, Figure S1: ^1^H NMR spectrum of C_8_MIMC in CDCl_3_; Figure S2: ^1^H NMR spectrum of C_14_MIMCl in CDCl_3_; Figure S3: The TEM images of C_8_MIM-MSNs (**a**), C_14_MIM-MSNs (**b**) and C_18_MIM-MSNs (**c**); Figure S4: Size distributions of MIMs as measured by dynamic light scattering in water; Scheme S1: Schematic illustration of the synthesis procedure of MSNs; Table S1: The statistical analysis of the reusabilities of MIMs.

## References

[1] He X., Chen Y., Wang K., Wu P., Gong P., Huo H. (2007). Selective separation of proteins with pH-dependent magnetic nanoadsorbents. Nanotechnology.

[2] MacCallum R.M., Martin A.C.R., Thornton J.M. (1996). Antibody-antigen interactions: Contact analysis and binding site topography. J. Mol. Biol..

[3] Cheong W.J., Ali F., Choi J.H., Lee J.O., Sung K.Y. (2013). Recent applications of molecular imprinted polymers for enantio-selective recognition. Talanta.

[4] Martín-Esteban A. (2013). Molecularly-imprinted polymers as a versatile, highly selective tool in sample preparation. TrAC Trends Anal. Chem..

[5] Qian L., Hu X., Guan P., Wang D., Li J., Du C., Song R., Wang C., Song W. (2015). The effectively specific recognition of bovine serum albumin imprinted silica nanoparticles by utilizing a macromolecularly functional monomer to stabilize and imprint template. Anal. Chim. Acta.

[6] Li S., Cao S., Whitcombe M.J., Piletsky S.A. (2014). Size matters: Challenges in imprinting macromolecules. Prog. Polym. Sci..

[7] Nishino H., Huang C.S., Shea K.J. (2006). Selective protein capture by epitope imprinting. Angew. Chem. Int. Ed..

[8] Tai D.F., Lin C.Y., Wu T.Z., Chen L.K. (2005). Recognition of dengue virus protein using epitope-mediated molecularly imprinted film. Anal. Chem..

[9] Yang Y.Q., He X.W., Wang Y.Z., Li W.Y., Zhang Y.K. (2014). Epitope imprinted polymer coating CdTe quantum dots for specific recognition and direct fluorescent quantification of the target protein bovine serum albumin. Biosens. Bioelectron..

[10] Rachkov A., Minoura N. (2000). Recognition of oxytocin and oxytocin-related peptides in aqueous media using a molecularly imprinted polymer synthesized by the epitope approach. J. Chromatogr. A.

[11] Shi Y., Wan Y., Zhao D. (2011). Ordered mesoporous non-oxide materials. Chem. Soc. Rev..

[12] Taguchi A., Schüth F. (2005). Ordered mesoporous materials in catalysis. Microporous Microporous Mater..

[13] Li W., Zhao D. (2013). An overview of the synthesis of ordered mesoporous materials. Chem. Commun..

[14] Gao Z.M., Yang X.Q., Wu N.N., Wang L.J., Wang J.M., Guo J., Yin S.W. (2014). Protein-based pickering emulsion and oil gel prepared by complexes of zein colloidal particles and stearate. J. Agric. Food Chem..

[15] Chen Y., Li X., Yin D., Li D., Bie Z., Liu Z. (2015). Dual-template docking oriented molecular imprinting: A facile strategy for highly efficient imprinting within mesoporous materials. Chem. Commun..

[16] Chen J., Lei S., Xie Y., Wang M., Yang J., Ge X. (2015). Fabrication of high-performance magnetic lysozyme-imprinted microsphere and its NIR-responsive controlled release property. ACS Appl. Mater. Interfaces.

[17] Yue Q., Li J., Luo W., Zhang Y., Elzatahry A.A., Wang X., Wang C., Li W., Cheng X., Alghamdi A. (2015). An interface coassembly in biliquid phase: Toward core–shell magnetic mesoporous silica microspheres with tunable pore size. J. Am. Chem. Soc..

[18] Hudson S., Cooney J., Magner E. (2008). Proteins in mesoporous silicates. Angew. Chem. Int. Ed..

[19] Zhou Y., Antonietti M. (2003). A novel tailored bimodal porous silica with well-defined inverse opal microstructure and super-microporous lamellar nanostructure. Chem. Commun..

[20] Žilková N., Zukal A., Čejka J. (2006). Synthesis of organized mesoporous alumina templated with ionic liquids. Microporous Microporous Mater..

[21] Kuang D., Brezesinski T., Smarsly B. (2004). Hierarchical porous silica materials with a trimodal pore system using surfactant templates. J. Am. Chem. Soc..

[22] Zhang J., Ma Y., Shi F., Liu L., Deng Y. (2009). Room temperature ionic liquids as templates in the synthesis of mesoporous silica via a sol-gel method. Microporous Microporous Mater..

[23] Lofgreen J.E., Ozin G.A. (2014). Controlling morphology and porosity to improve performance of molecularly imprinted sol-gel silica. Chem. Soc. Rev..

[24] Sprynskyy M., Kowalkowski T., Tutu H., Cukrowska E.M., Buszewski B. (2015). Ionic liquid modified diatomite as a new effective adsorbent for uranium ions removal from aqueous solution. Colloids Surf. A Physicochem. Eng. Asp..

[25] Shen G., Zhang X., Shen Y., Zhang S., Fang L. (2015). One-step immobilization of antibodies for α-1-fetoprotein immunosensor based on dialdehyde cellulose/ionic liquid composite. Anal. Biochem..

[26] Zhang N., Hu X., Guan P., Du C., Li J., Qian L., Zhang X., Ding S., Li B. (2017). Preparation of protein imprinted microspheres using amphiphilic ionic liquid as stabilizer and emulsifier via miniemulsion polymerization. Chem. Eng. J..

[27] Trewyn B.G., Whitman C.M., Lin V.S.Y. (2004). Morphological control of room-temperature ionic liquid templated mesoporous silica nanoparticles for controlled release of antibacterial agents. Nano Lett..

[28] Hartmann M., Jung D. (2010). Biocatalysis with enzymes immobilized on mesoporous hosts: The status quo and future trends. J. Mater. Chem..

[29] Wu X., Hou M., Ge J. (2015). Metal-organic frameworks and inorganic nanoflowers: A type of emerging inorganic crystal nanocarrier for enzyme immobilization. Catal. Sci. Technol..

[30] Ding S., Hu X., Guan P., Zhang N., Li J., Gao X., Zhang X., Ding X., Du C. (2017). Preparation of surface-imprinted microspheres using ionic liquids as novel cross-linker for recognizing an immunostimulating peptide. J. Mater. Sci..

[31] Du C., Hu X., Guan P., Gao X., Song R., Li J., Qian L., Zhang N., Guo L. (2016). Preparation of surface-imprinted microspheres effectively controlled by orientated template immobilization using highly cross-linked raspberry-like microspheres for the selective recognition of an immunostimulating peptide. J. Mater. Chem. B.

[32] Tominaga Y., Kubo T., Kaya K., Hosoya K. (2009). Effective Recognition on the surface of a polymer prepared by molecular imprinting using ionic complex. Macromolecules.

[33] Putra E.K., Pranowo R., Sunarso J., Indraswati N., Ismadji S. (2009). Performance of activated carbon and bentonite for adsorption of amoxicillin from wastewater: Mechanisms, isotherms and kinetics. Water Res..

[34] Qian L., Sun J., Hou C., Yang J., Li Y., Lei D., Yang M., Zhang S. (2017). Immobilization of BSA on ionic liquid functionalized magnetic Fe_3_O_4_ nanoparticles for use in surface imprinting strategy. Talanta.

[35] Gao X., Hu X., Guan P., Du C., Ding S., Zhang X., Li B., Wei X., Song R. (2016). Synthesis of core–shell imprinting polymers with uniform thin imprinting layer via iniferter-induced radical polymerization for the selective recognition of thymopentin in aqueous solution. RSC Adv..

[36] Tamahkar E., Kutsal T., Denizli A. (2015). Surface imprinted bacterial cellulose nanofibers for cytochrome c purification. Process Biochem..

[37] Zhang X., Zhang N., Du C., Guan P., Gao X., Wang C., Du Y., Ding S., Hu X. (2017). Preparation of magnetic epitope imprinted polymer microspheres using cyclodextrin-based ionic liquids as functional monomer for highly selective and effective enrichment of cytochrome c. Chem. Eng. J..

[38] Guo T., Deng Q., Fang G., Liu C., Huang X., Wang S. (2015). Molecularly imprinted upconversion nanoparticles for highly selective and sensitive sensing of cytochrome c. Biosens. Bioelectron..

