# Supplementary material for: Preparation of Molecularly Imprinted Mesoporous Materials for Highly Enhancing Adsorption Performance of Cytochrome C
Source: Polymers (Basel). 2018 Mar 10;10(3):298. doi: 10.3390/polym10030298 (PMC6414899; doi:10.3390/polym10030298)
Supplement: Supplementary file 1 [file polymers-10-00298-s001.doc]

**Supplementary Materials:**

Preparation of Molecularly Imprinted Mesoporous Materials for Highly Enhancing Adsorption Performance of Cytochrome C

**Zhiling Li^1^, Ping Guan^1^,*, Xiaoling Hu^1^, Shichao Ding^1^, Yuan Tian^1^, Yarong Xu^1^, Liwei Qian^2^,***

^1^ Key Laboratory of Space Applied Physics and Chemistry of Ministry of Education,
Department of Applied Chemistry, School of Nature and Applied Science,
Northwestern Polytechnical University, Xi’an 710072, China; lizhiling1993@mail.nwpu.edu.cn (Z.L.);
huxl@nwpu.edu.cn (X.H.); dingshichao1992@163.com (S.D.); summer940324@163.com (Y.T.); xuyarong@mail.nwpu.edu.cn (Y.X.)

^2^ College of Bioresources Chemical and Materials Engineering, Shaanxi University of Science and Technology, Xi’an 710021, China

***** Correspondence: guanping1113@nwpu.edu.cn (P.G.); qianliwei@mail.nwpu.edu.cn (L.Q.); Tel.: +86-029-8843-1664 (P.G.); +86-029-8669-0006 (L.Q.)

**Experimental**

*Synthesis of amphiphilic ionic liquids C_n_MIMCl (n = 8, 14)*

The synthesis of C_8_MIMCl and C_14_MIMCl was same as C_18_MIMCl. 1-Methylimidazole (0.1mol) was mixed with 0.1mol of 1-Chlorooctane and 1-Chlorotetradecane, respectively. The mixture was put into a 250 mL flask, refluxed at 90 ^o^C for 24 h. When cooling to room temperature, a white solid was obtained. The product was further purified by recrystallization in THF. After washing several times with THF, the resulting white powder were collected by filtration and dried under vacuum at room temperature. The structure of C_n_MIMCl is shown in Figure S1 and Figure S2.

*Synthesis of C_n_MIM-MSN materials*

In a typical procedure for the synthesis of the C_n_MIM-MSN materials, tetraethylorthosilicate (TEOS) was used as the silica source and C_n_MIMCl as the surfactant. Firstly, a selected C_n_MIMCl was dissolved in 480 mL of 15 mM NaOH (aq) under mild magnetic stirring. The amount of C_n_MIMCl was calculated by the critical micelle concentration (CMC). After homogenization of the mixture, TEOS was added dropwise at 80 ^o^C and stirred for 2 h to yield the desired C_n_MIM-MSN materials. Then the C_n_MIM ILs were removed from mesopores by Soxhlet extraction. The egression of ILs from the C_n_MIM-MSN materials was traced by UV-vis absorption. Finally, the final product was dried under vacuum until weight did not change.

**Adsorption experiments**

The adsorption capacities *Q_e_* (mg g^-1^) of the MIMs (NIMs) for Cyt c were calculated using the following formula:

$Qe=\frac{(C0-Ce)V}{m}$Equation S1

where *C_0_* (mg mL^-1^) is the initial concentration of the Cyt c buffer solution, *C_e_* (mg mL^-1^) is the concentration of Cyt c buffer solution in the equilibrium solution, *V* (mL) is the volume of the Cyt c buffer solution and *m* (g) is the mass of MIMs or NIMs.

The recognition selectivity was evaluated by imprinting factor (*IF*), which were defined as follows:

Equation S2

where *Q*_MIMs_ and *Q*_NIMs_ are the adsorption capacities of Cyt c, respectively.

For further investigate the template affinity of MIMs and NIMs, adsorption isotherms are described by the Langmuir model and Freundlich model,

Equation S3

$Qe=KFC_{e}^{1/n}$ Equation S4

where *C_e_* (mg mL^–1^) is the equilibrium concentration of IHHC in equilibrium solution, *Q_e_* and *Q*_max_ (mg g^–1^) are the experimental adsorption capacity and maximum theoretical adsorption capacity of the adsorbents towards Cyt c, respectively. And *K_L_* (mL mg^–1^) is the Langmuir adsorption equilibrium constant related to the affinity of the active sites. Moreover, the *K_F_* (mg g^-1^) and *n* are the Freundlich constants.

To further investigate the adsorption kinetics, we applied the pseudo-first-order kinetic model and pseudo-second-order kinetic model to fit the kinetic data. The pseudo-first-order and pseudo-second-order are expressed as followed,

Equation S5

Equation S6

where *Q_t_* (mg g^−1^) and *Q_e_* (mg g^−1^) are the adsorption amount for Cyt c at any time t and at the equilibrium, respectively. *K_1t_* (min^−1^) and *K_2_* (g mg^−1^ min^−1^) are rate constants of the pseudo-first-order and pseudo-second-order models.

**Results and discussion**

**
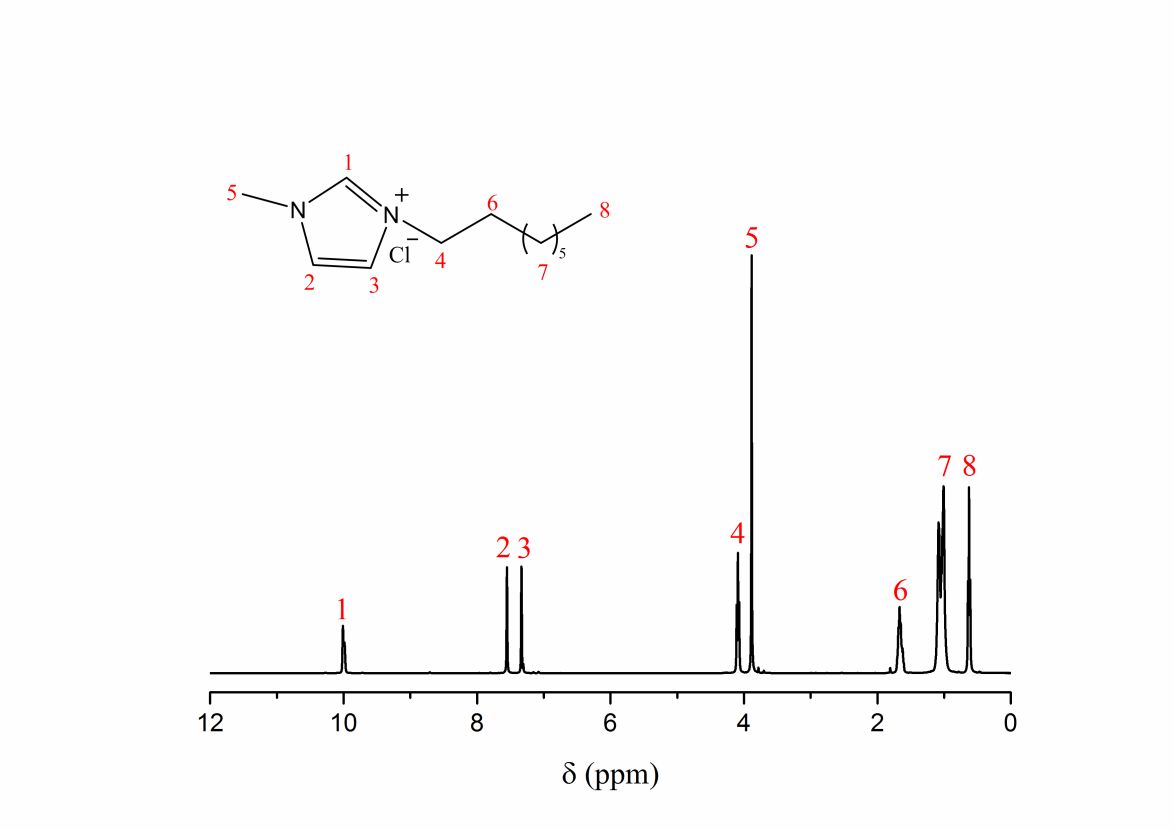
**

**Figure S1.** ^1^H NMR spectrum of C_8_MIMC in CDCl_3_.


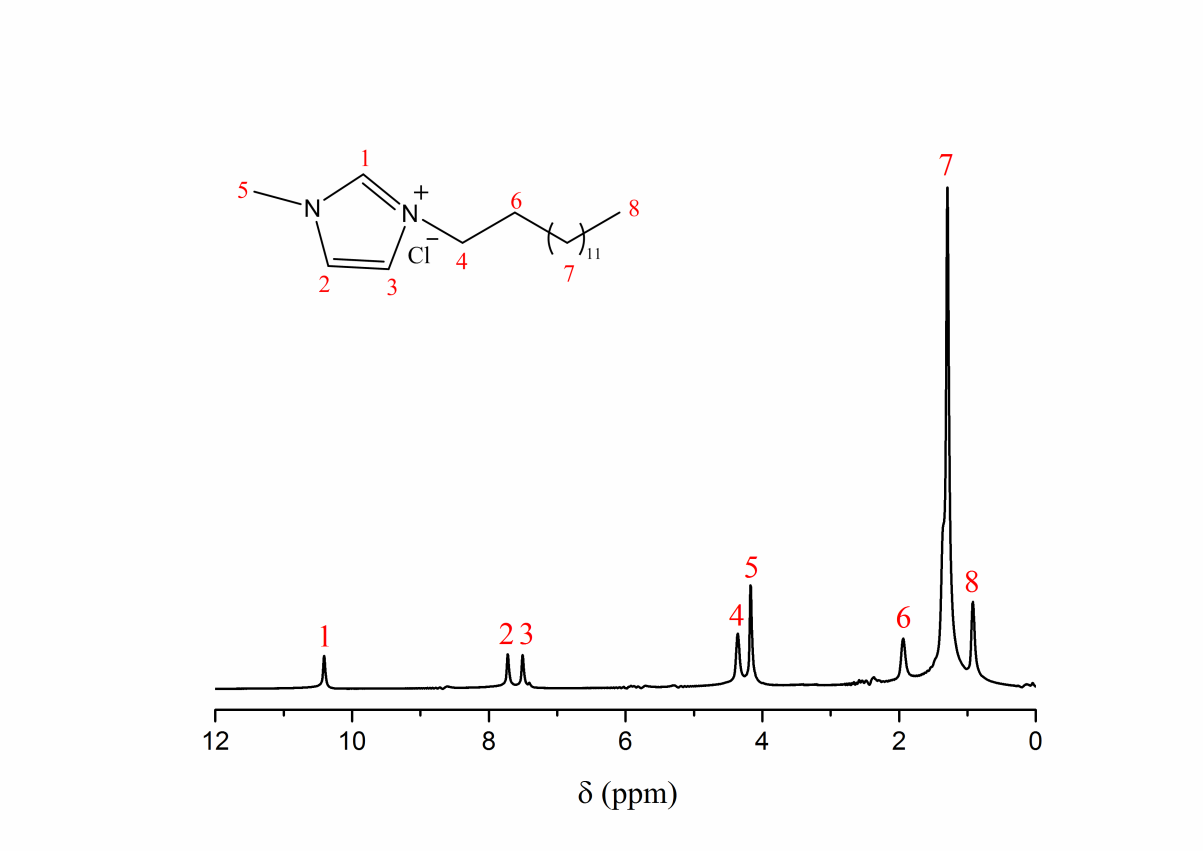


**Figure S2.** ^1^H NMR spectrum of C_14_MIMCl in CDCl_3_.


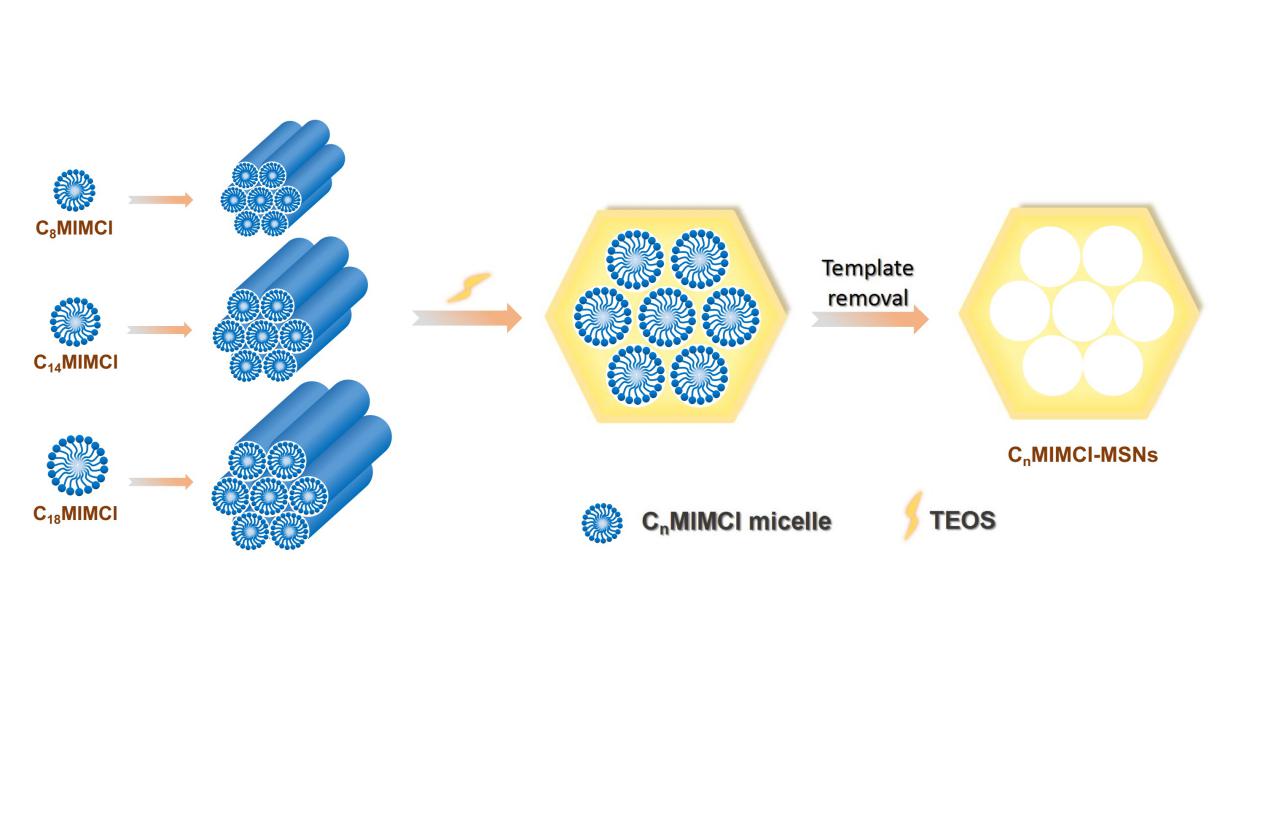


**Scheme S1.** Schematic illustration of the synthesis procedure of MSNs


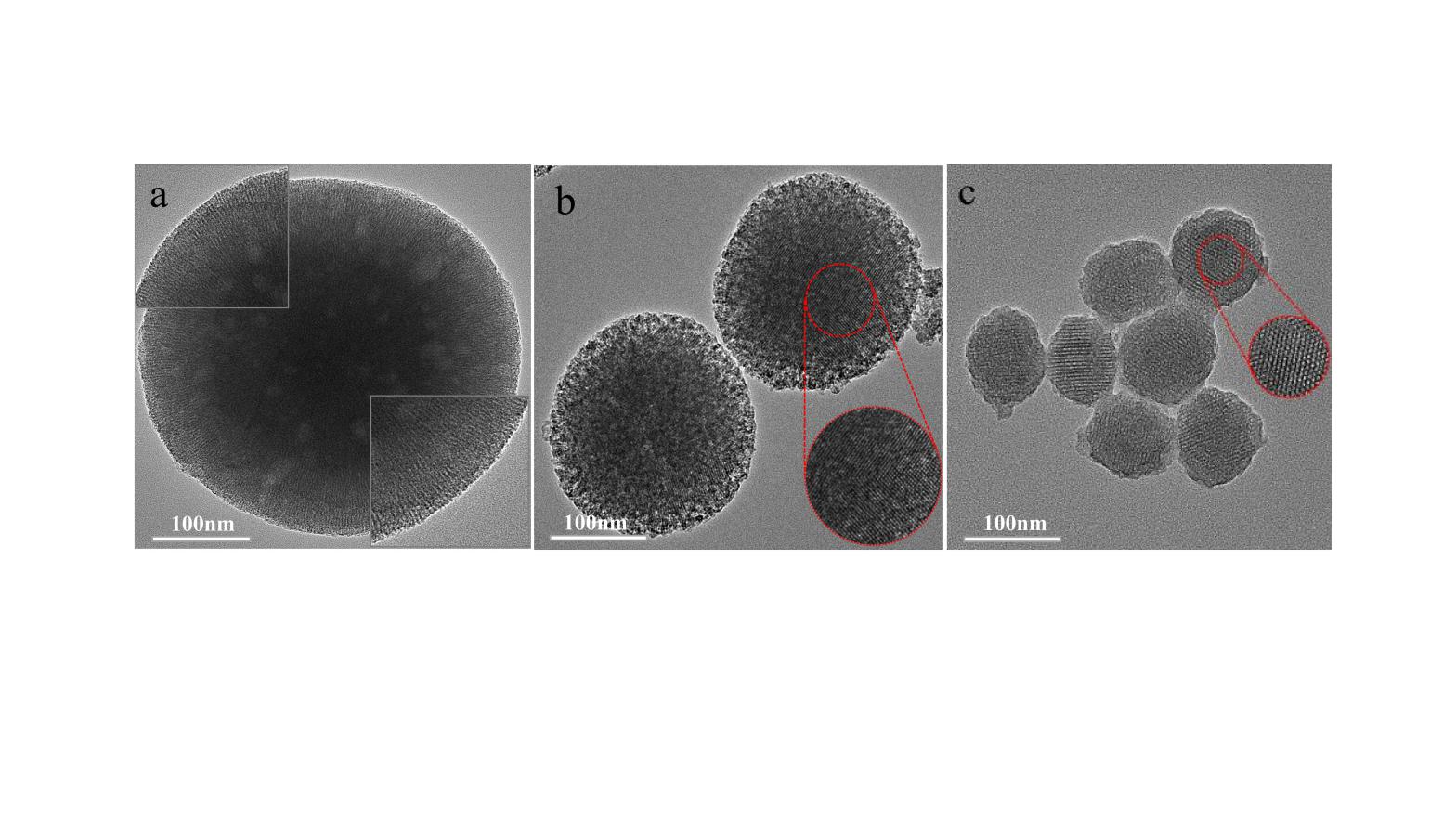


**Figure S3.** The TEM images of C_8_MIM-MSNs (a), C_14_MIM-MSNs (b) and C_18_MIM-MSNs (c).


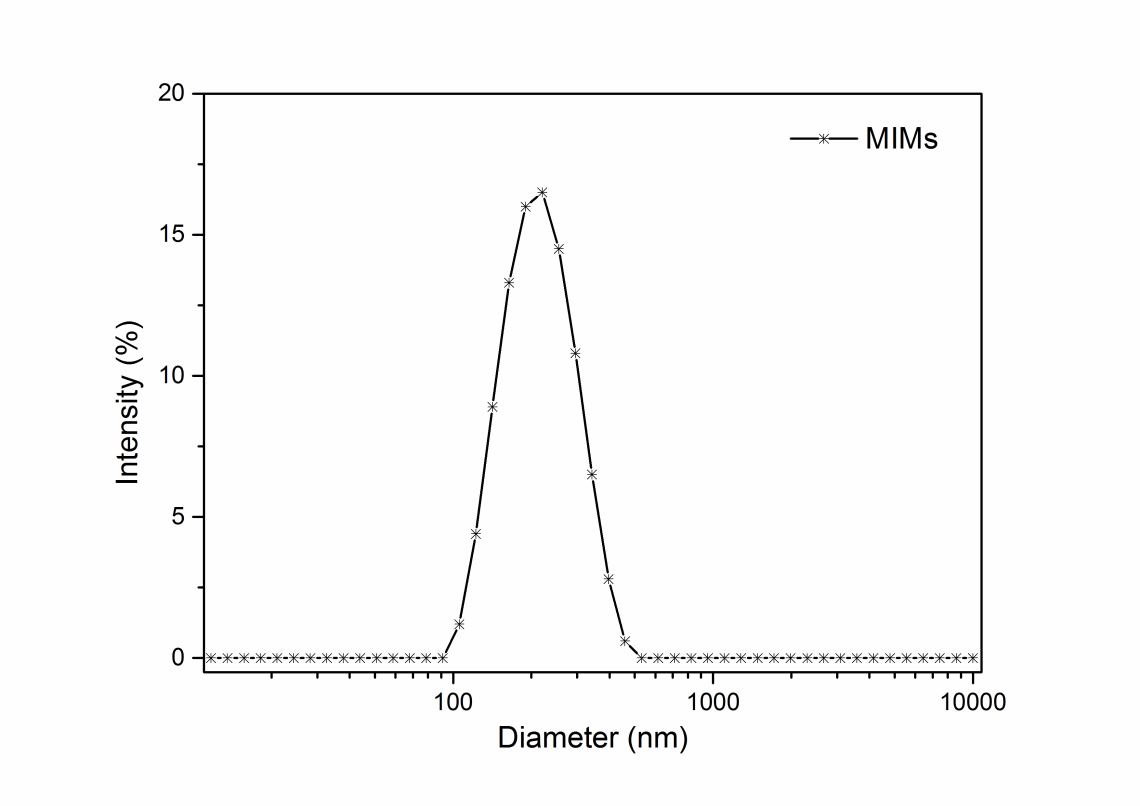


**Figure S4.** Size distributions of MIMs as measured by dynamic light scattering in water.

**Table S1**. The statistical analysis of the reusabilities of MIMs.

| **Cycle number** | ***Q* (mg g^-1^)** | ***Q*_1_ (mg g^-1^)** |
| --- | --- | --- |
| 1 | 86.47 | 0 |
| 2 | 82.16 | 4.31 |
| 3 | 78.56 | 3.60 |
| 4 | 78.79 | -0.03 |
| 5 | 75.56 | 3.23 |
| 6 | 73.04 | 2.52 |

*Q*_1_ is the decreased adsorption amount of each elution step.
